# Combining Results from Distinct MicroRNA Target Prediction Tools Enhances the Performance of Analyses

**DOI:** 10.3389/fgene.2017.00059

**Published:** 2017-05-16

**Authors:** Arthur C. Oliveira, Luiz A. Bovolenta, Pedro G. Nachtigall, Marcos E. Herkenhoff, Ney Lemke, Danillo Pinhal

**Affiliations:** ^1^Laboratory of Genomics and Molecular Evolution, Department of Genetics, Institute of Biosciences of Botucatu, São Paulo State Univesity (UNESP)Botucatu, Brazil; ^2^Laboratory of Bioinformatics and Computational Biophysics, Department of Physics and Biophysics, Institute of Biosciences of Botucatu, São Paulo State Univesity (UNESP)Botucatu, Brazil

**Keywords:** *in silico* prediction, TargetScan, miRanda-mirSVR, Pita, RNA22, non-coding RNA, bioinformatics

## Abstract

Target prediction is generally the first step toward recognition of bona fide microRNA (miRNA)-target interactions in living cells. Several target prediction tools are now available, which use distinct criteria and stringency to provide the best set of candidate targets for a single miRNA or a subset of miRNAs. However, there are many false-negative predictions, and consensus about the optimum strategy to select and use the output information provided by the target prediction tools is lacking. We compared the performance of four tools cited in literature—TargetScan (TS), miRanda-mirSVR (MR), Pita, and RNA22 (R22), and we determined the most effective approach for analyzing target prediction data (individual, union, or intersection). For this purpose, we calculated the sensitivity, specificity, precision, and correlation of these approaches using 10 miRNAs (miR-1-3p, miR-17-5p, miR-21-5p, miR-24-3p, miR-29a-3p, miR-34a-5p, miR-124-3p, miR-125b-5p, miR-145-5p, and miR-155-5p) and 1,400 genes (700 validated and 700 non-validated) as targets of these miRNAs. The four tools provided a subset of high-quality predictions and returned few false-positive predictions; however, they could not identify several known true targets. We demonstrate that union of TS/MR and TS/MR/R22 enhanced the quality of *in silico* prediction analysis of miRNA targets. We conclude that the union rather than the intersection of the aforementioned tools is the best strategy for maximizing performance while minimizing the loss of time and resources in subsequent *in vivo* and *in vitro* experiments for functional validation of miRNA-target interactions.

## Introduction

MicroRNAs (miRNAs) are a large class of small non-coding RNAs [∼22 nucleotides (nts)] that post-transcriptionally regulate gene expression. They were first identified in the context of *Caenorhabditis elegans* development (Lee et al., 1993), and they are now known to regulate most biological process in animals, plants, and even certain viruses (Lee et al., 1993; Sunkar et al., 2005; Jia et al., 2008). Their function ranges from cellular proliferation and differentiation to response to environmental stimuli and diseases such as cancer (Qiu et al., 2012; Shenoy and Blelloch, 2014; Reddy, 2015). Therefore, identification of their target genes is important for understanding their role in the complex biological regulatory pathways regulated by miRNA-target interactions.

In animals, a sequence of approximately seven nts in the 5′ region of the miRNA (ranging from nts 2 to 8), known as the seed region, guides the miRNA to its target mRNA. Five types of perfect Watson–Crick pairing of seed matches have been described so far, namely, 8-mer, 7-mer-m8, 7-mer-A1, 6-mer, and offset-6-mer in the descending order of the strength of their matches (Agarwal et al., 2015). The 8-mer site is a perfect match for nts 2–8, with an adenine at relative nt 1 in the mRNA. The 7-mer-m8 is a perfect match for nts 2–8, whereas the 7-mer-A1 is a perfect match for nts 2–7, with an adenine at relative nt 1 in the mRNA. The weaker 6-mer and offset-6-mer are perfect matches for nts 2–7 and 3–8, respectively. The adenosine at relative nt position 1 of the mRNA supports the miRNA-mediated regulation, even if the opposing nt does not form a Watson–Crick pairing (Baek et al., 2008). In addition to the seed-based interactions, recent studies also reported miRNA regulation through non-seed interactions, demonstrating that the 3′ region of the miRNA transcript might be equally important as the seed sequence for securing target recognition (Tay et al., 2008; Nelson et al., 2011; Chi et al., 2012; Clarke et al., 2012; Broughton et al., 2016).

Irrespective of seed or non-seed match, miRNA pairing is largely prevalent with elements at the 3′ untranslated region (UTR) of target genes. However, studies have identified miRNA pairing to sites outside the 3′UTR, both in the coding region (Tay et al., 2008; Schnall-Levin et al., 2010; Gartner et al., 2013; Hausser et al., 2013) and in the 5′UTR (Lytle et al., 2007; Orom et al., 2008; Devlin et al., 2010; Zhou and Rigoutsos, 2014) of the mRNA. Such findings showed that although the 3′UTR is the main site of miRNA pairing, the whole mRNA transcript should be inspected when predicting miRNA-target interactions.

Currently, several *in silico* tools are available for identifying putative miRNA targets. The main parameters used by these tools can be gathered and divided into three groups: duplex features, local context features, and global context features (Betel et al., 2010). Duplex features encompass seed match, 3′ contribution, seed pairing stability (SPS; Betel et al., 2010), heteroduplex free energy, and *p*-value (Miranda et al., 2006). These parameters evaluate the hybridization of the miRNA to its target gene. Seed match evaluates the number of nts that can bind to the mRNA target in the seed region. The 3′ contribution evaluates the possibility of binding at the 3′ position of the miRNA (Witkos et al., 2011). The SPS evaluates the types of nts compose the seed region (Garcia et al., 2011). The heteroduplex free energy evaluates whether the minimum free energy between the miRNA and its target is sufficient to establish hybridization, and the *p*-value evaluates whether the probability of a selected interaction has been predicted by chance.

Local context features include mRNA sequence properties that directly influence target recognition, such as site accessibility (SA) and presence of flanking AU. SA evaluates the capacity of the mRNA to unfold into a potential secondary structure in the region containing the miRNA cognate sequence, which is known as the miRNA recognition element (MRE; Kertesz et al., 2007). The flanking AU corresponds to the number of A and U nts flanking the MRE region. High concentrations of flanking A and U nts enhance miRNA regulation (Grimson et al., 2007).

Global context features aggregate mRNA sequence properties with indirect influence on target recognition, such as whole transcript length, 3′UTR length, transcriptome abundance, pairing position at the 3′UTR, and sequence conservation. Sequence length evaluates the total length of the string analyzed, since the chances of false prediction increases with target length (Miranda et al., 2006). The 3′UTR length, as the name suggests, evaluates the length of the 3′UTR of the potential miRNA targets, since larger 3′UTRs are regulated more stringently than shorter ones (Sandberg et al., 2008). Transcriptome abundance evaluates the number of MREs of a miRNA within the transcriptome. Pairing position evaluates the position of the MRE within the 3′UTR, because MREs near the ends of the 3′UTR have stronger regulatory potential (Grimson et al., 2007). Finally, sequence conservation evaluates the extent of conservation of the MREs among species. Together, all these binding metrics decisively regulate the determination of potential miRNA-target pairs.

Despite the availability of several target prediction tools that use distinct parameters and strategies to search for putative targets, consensus about the best tool is lacking. In fact, experimental validation (the usual step after target prediction) has revealed many false-negative predictions, implying that further improvement of prediction tools is required. To circumvent this caveat, researchers use diverse strategies for determining putative miRNA targets, including intersection and union of predictions. However, this approach is being used indiscriminately, without well-defined criteria and rigorous comparative tests to assess the performance of the prediction strategies. Thus, whether union or intersection of results obtained from multiple tools improves the overall quality of target prediction is yet unknown.

Here, we compared the performance of four widely used target prediction tools to identify the strategy that best predicts miRNA targets. Our results would assist researchers in selecting the correct candidates for subsequent experimental validation of miRNA-target interactions.

## Materials and Methods

### Target Prediction Tools Data

We used TargetScan (TS), miRanda-mirSVR (MR), Pita (PT), and RNA22 (R22) pre-computed predictions, which are freely available online. TS, MR, and PT consider seed-based interactions in the 3′UTR, whereas R22 also considers non-seed based interactions (full-length matches) in the whole transcript.

These tools were selected based on their recognized popularity among researchers and the presence of an update policy (i.e., data is updated when new miRNAs and/or parameters are reported). We exclusively used the best predictions from each database to maximize the quality of predictions (summarized in **Table 1**). In detail, the best predictions were those with conserved sites for TS. TS considers different cutoffs for conservation, according to seed match; for example, it is ≥0.8 for site 8-mer, ≥1.3 for site 7-mer-m8, and ≥1.6 for site 7-mer-A1, whereas sites 6-mer and offset 6-mer are always classified as non-conserved^1^. Best predictions of MR present good mirSVR score (≤ -0.1) and conserved sites (PhastCOns score >0.57; Betel et al., 2008). PT ranks those with seed match to 7- or 8-mer and conservation score ≥0.9^2^ as best predictions, whereas R22 best predictions comprise those with base pair minimum value of 12, folding energy max value of -12 kcal/mol, max *p*-value of 0.1, and miRbase 21/Ensembl 78 databases.

**Table 1:** Summary of the target prediction tools analyzed.

	TargetScan	miRanda-mirSVR	Pita	RNA22
Website	targetscan.org	microrna.org	genie.weizmann.ac.il	https://cm.jefferson. edu/rna22/
Version	v7.1 (06/2016)	V3.3a (08/2010)	V6 (08/2008)	V2 (04/2015)
Predictions downloaded	Conserved sites	Good mirSVR score, Conserved miRNA	Seed 7- or 8-mer and conservation score 0.9 or higher	Base pair: >12 Folding energy: ≤ -12 kcal/mol *p*-value: ≤ -0.1 miRbase 21/Ensembl 78
Reference	Lewis et al., 2005	Enright et al., 2003	Kertesz et al., 2007	Miranda et al., 2006


Gene names predicted were converted to the Ensembl gene ID to standardize the annotations from all tools. We also combined the outputs of the tools to evaluate union and intersection approaches. The unions tested were TS + MR + PT + R22, TS + MR + PT, TS + MR + R22, TS + PT + R22, MR + PT + R22, TS + MR, TS + PT, TS + R22, MR + PT, MR + R22, and PT + R22. The intersections tested were TS + MR + PT + R22, TS + MR + PT, TS + MR + R22, TS + PT + R22, MR + PT + R22, TS + MR, TS + PT, TS + R22, MR + PT, MR + R22, PT + R22, and majority vote. The majority vote consists of counting any target that was predicted by at least two of the four tools.

### Performance Evaluation

In order to evaluate the performance of each tool and the combinatorial method, we downloaded the validated miRNA target dataset for the human genome from miRTarBase^3^; v6 – 09/2015; Chou et al., 2016). Then, we selected 10 miRNAs with the highest number of validated targets, including miR-155-5p (224 validated targets), miR-145-5p (129 validated targets), miR-21-5p (115 validated targets), miR-34a-5p (101 validated targets), miR-29a-3p (96 validated targets), miR-125b-5p (83 validated targets), miR-124-3p (83 validated targets), miR-24-3p (83 validated targets), miR-17-5p (74 validated targets), and miR-1-3p (73 validated targets). This analysis was limited to these 10 miRNAs due to the few number of validated targets available to the other miRNAs, which inclusion would prejudice the power of the statistical analysis. We analyzed only “strong validations” assigned by miRTarBase, which refer to miRNA-target interactions validated using reporter assays, western blot and/or quantitative polymerase chain reaction (qPCR). We did not include “less strong validations,” such as those reported using microarray, pSILAC, and next generation sequencing (NGS)-based experiments (e.g., Ago HITS-CLIP, degradome-seq, CLASH, PAR-CLIP, and iPAR-CLIP) to enforce maximum stringency.

We calculated the sensitivity, specificity, precision, and performance of each target prediction tool and their combinations. The performance was calculated using Matthews correlation coefficient (MCC):

$$\text{Sensitivity=}\frac{\text{TP}}{\text{TP+FN}}$$

$$\text{Specificity=}\frac{\text{TN}}{\text{TN+FP}}$$

$$\text{Precision=}\frac{\text{TP}}{\text{TP+FP}}$$

$$\text{MCC=}\frac{\text{TP*TN-FP*FN}}{\sqrt{(\text{TP+FP})\left(\text{TP+FN}\right)\left(\text{TN+FP}\right)\left(\text{TN+FN}\right)}}$$

where TP (true positive) is the number of validated targets predicted, FN (false negative) is the number of validated targets not predicted, FP (false positive) is the number of predicted targets that were not validated, and TN (true negative) is the number of genes that were neither predicted nor validated. Sensitivity and specificity are mathematical functions that measure the quality of binary classifications. Since *in silico* target prediction tools are binary classifiers, these two functions can be used to evaluate the quality of each tool. Sensitivity measures a tool’s ability to identify bona fide miRNA targets, while specificity measures the capacity of the tool to correctly exclude a gene target that is not regulated by the miRNA (Parikh et al., 2008).

Knowledge about the proportion of true predictions within the total number of miRNA targets predicted is also important. Therefore, precision is calculated to evaluate the number of true targets among all predicted targets (Powers, 2007). Finally, MCC can combine all these values to generate a unique comparable number. MCC is a recognized measure that is used to evaluate the quality of binary classifiers (i.e., true targets/false targets), and it is often used to classify miRNA target prediction tools (Bandyopadhyay and Mitra, 2009; Fan and Kurgan, 2015).

The sensitivity, specificity, and precision values range from 0 to 1, with near zero values indicating low quality results and values near one representing high quality results. MCC ranges from -1 to 1, which represent low quality and high quality predictions, respectively. Values near zero indicate predictions that are similar to random predictions. To calculate these values, we randomly selected 70 validated targets as the true set and 70 non-validated genes as the negative set for each miRNA, with reposition. This generated 1,400 genes (700 true and 700 false) for each replicate (*N* = 5). Finally, the average of the five replicates was calculated and subjected to statistical analysis (see Supplementary Table S1 for individual values from each replicate and each miRNA).

To confirm whether the specificity values of the tools were biased due to the lack of false predictions in literature, we performed a control test by predicting putative targets in a random strings analysis. Toward this objective, we generated four different groups with 1,000 random strings each, totaling to 4,000 random strings. Groups of variable length (500, 1,000, 2,500, and 5,000 nts each) were tested because length highly influences the chances of false prediction (Miranda et al., 2006). Then, we downloaded the source code of each tool and locally ran the predictions of the ten miRNAs with the same parameters used for the best predictions of the pre-computed data, with the exception of the “conservation score” for TS, MR, and PT, and the “mirSVR score” for MR, which was not available on the miRanda source code (**Table 1**).

### Statistical Analysis

To compare the performance of each tool and the combinatorial method, we used one-way analysis of variance (ANOVA) and the Tukey test for multiple comparisons (*p*-value < 0.05) since the data presented a Gaussian distribution.

## Results

### Target Prediction Outputs

**FIGURE 1:**
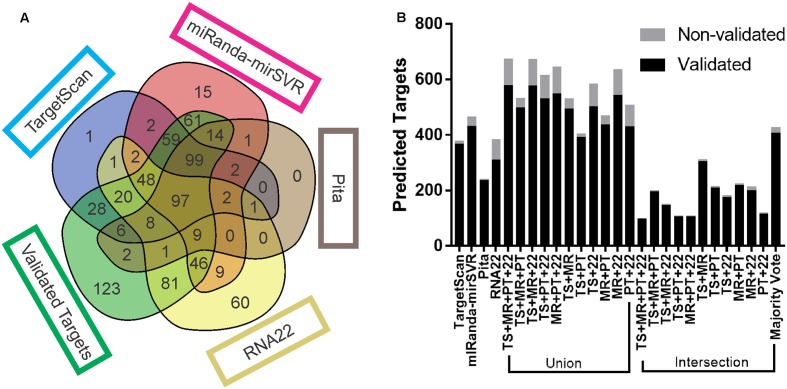
**Target prediction output comparison.** **(A)** Venn diagram of the number of validated and non-validated targets predicted by each tool, as well as the number targets that were not predicted. **(B)** Total number of validated and non-validated targets predicted by each tool and combinatorial approach. Venn diagrams from all replicates are available at Supplementary Data Sheet S1.

Each target prediction tool noticeably generated different results. TS and MR had the highest number of mutual targets (310 predicted and 303 validated). The number of targets predicted by TS, MR, and PT (99 validated and 2 non-validated) was equivalent to the number predicted by all tools together (97 validated and 2 non-validated) (**Figure 1A**). MR itself predicted the highest number of targets among all the tools, of which 433 were validated and 33 were non-validated. PT predicted the lowest number (234 validated and 6 non-validated), TS predicted 366 validated and 11 non-validated targets, and R22 predicted 325 validated and 60 non-validated. R22 predicted the highest number of validated targets not identified by any other tool (81; with 60 non-validated), followed by MR (61 validated and 15 non-validated), TS (28 validated and 1 non-validated), and PT (2 validated and 0 non-validated). Interestingly, of the 81 validated targets predicted exclusively by R22, 56 possessed non-canonical sites, 34 of which had sites only outside the 3′UTR (either seed-based or full-length), and 22 targets with sites inside the 3′UTR but with a mismatch in the seed region.

The majority of intersections consistently returned a lower number of predicted targets than any other approach, with the exception of the intersection of TS + MR (304 validated and 7 non-validated) and majority vote (406 validated and 19 non-validated), which predicted more targets than PT. All the unions predicted more targets than any other approach (**Figure 1B**), except for TS + PT (390 validated and 12 non-validated), which predicted less targets than MR and majority vote.

The four tools were able to recover much more true predictions than false predictions (366 validated and 11 non-validated for TS; 433 validated and 33 non-validated for MR; 234 validated and 6 non-validated for PT; 325 validated and 60 non-validated for R22). However, approximately 18% of the validated targets (123) were not predicted by any tool. Supplementary Table S2 shows the predicted targets of the 10 miRNAs by each tool.

### Sensitivity, Specificity, and Precision of the Methods

**Table 2** summarizes the sensitivity, specificity, and precision of all tools and methods. All methods showed striking specificity (>0.85) and precision (>0.80), but variable sensitivity. Considering the four tools individually, MR showed the highest sensitivity (0.62) and R22 showed the lowest specificity (0.89) and precision (0.81). TS and PT showed similar values of specificity and precision, but PT showed a significantly lower sensitivity.

**Table 2:** Sensitivity, specificity, and precision of the target prediction methods.

Method	Tool	Sensitivity	Specificity	Precision
Individual tool	TargetScan	0.524 ± 0.004	0.984 ± 0.005	0.971 ± 0.004
	miRanda-mirSVR	0.617 ± 0.012	0.954 ± 0.006	0.930 ± 0.010
	Pita	0.336 ± 0.009	0.992 ± 0.004	0.977 ± 0.011
	RNA22	0.336 ± 0.009	0.893 ± 0.019	0.805 ± 0.027
Union	TS + MR + PT + R22	0.825 ± 0.008	0.862 ± 0.020	0.857 ± 0.017
	TS + MR + PT	0.710 ± 0.006	0.949 ± 0.007	0.933 ± 0.009
	TS + MR + R22	0.822 ± 0.007	0.862 ± 0.020	0.857 ± 0.017
	TS + PT + R22	0.757 ± 0.038	0.879 ± 0.028	0.863 ± 0.023
	MR + PT + R22	0.784 ± 0.006	0.865 ± 0.019	0.853 ± 0.018
	TS + MR	0.706 ± 0.006	0.949 ± 0.008	0.932 ± 0.009
	TS + PT	0.558 ± 0.007	0.983 ± 0.005	0.970 ± 0.009
	TS + R22	0.716 ± 0.004	0.885 ± 0.21	0.862 ± 0.21
	MR + PT	0.624 ± 0.016	0.954 ± 0.006	0.931 ± 0.010
	MR + R22	0.773 ± 0.008	0.865 ± 0.019	0.851 ± 0.019
	PT + R22	0.613 ± 0.005	0.889 ± 0.020	0.847 ± 0.023
Intersection	TS + MR + PT + R22	0.139 ± 0.006	0.998 ± 0.003	0.984 ± 0.018
	TS + MR + PT	0.279 ± 0.014	0.994 ± 0.003	0.980 ± 0.010
	TS + MR + R22	0.201 ± 0.007	0.995 ± 0.005	0.976 ± 0.022
	TS + PT + R22	0.150 ± 0.006	0.997 ± 0.002	0.976 ± 0.016
	MR + PT + R22	0.151 ± 0.005	0.997 ± 0.003	0.978 ± 0.018
	TS + MR	0.435 ± 0.013	0.989 ± 0.004	0.976 ± 0.009
	TS + PT	0.298 ± 0.012	0.993 ± 0.003	0.978 ± 0.009
	TS + R22	0.249 ± 0.006	0.992 ± 0.004	0.969 ± 0.014
	MR + PT	0.312 ± 0.013	0.993 ± 0.003	0.977 ± 0.010
	MR + R22	0.285 ± 0.008	0.981 ± 0.001	0.940 ± 0.005
	PT + R22	0.164 ± 0.004	0.996 ± 0.003	0.974 ± 0.018
	Majority vote	0.581 ± 0.11	0.973 ± 0.005	0.955 ± 0.008


*TS, TargetScan; MR, miRanda-mirSVR; PT, Pita; R22, RNA22.*

The union of the four tools undoubtedly returned the best sensitivity, with TS + MR + PT + R22 and TS + MR + R22 returning values above 0.80. Interestingly, the increase in sensitivity had no negative impact in the specificity and precision indexes. By contrast, the intersection of tools resulted in low levels of sensitivity, except for the intersection of TS + MR and majority vote that showed higher sensitivity than PT alone. Overall, there was no improvement in specificity and precision upon using the intersection approach, with values closely resembling to those obtained by PT or TS alone.

For the random strings control analysis, the specificity of all tools decreased with increase in string length (**Table 3**), which corroborates the data from Miranda et al. (2006). When the results from all string lengths were summed, the tools had worse specificity values than their pre-computed data. However, the human 3′UTR ranges from 200 to 2,500 nts in length (average = 1,040 nts; Kotagama et al., 2015). Thus, when we summed the results only from 500, 1,000, and 2,500 nts, the specificity of TS, MR, and PT approached, while R22 equaled, to those observed in their pre-computed data. Therefore, these results suggest that the differences in specificities observed for TS, MR, and PT are more likely to be related to the lack of the conservation parameter (mirSVR score parameter for MR), which were not evaluated by R22, than to a bias in validation experiments.

**Table 3:** Specificity values of random string predictions.

Strings length	TargetScan	miRanda-mirSVR	Pita	RNA22
500 nt	0.9489	0.9446	0.8874	0.9338
1,000 nt	0.8966	0.8903	0.7917	0.8766
2,500 nt	0.7668	0.7550	0.5562	0.7466
5,000 nt	0.5846	0.5596	0.3081	0.6008
500 + 1,000 + 2,500 + 5,000	0.7992	0.7874	0.6359	0.7895
500 + 1,000 + 2,500	0.8708	0.8633	0.7451	0.8523


*Each length contains 1,000 strings with random sequence of ATCG nucleotides.*

### Evaluating the Performance of the Methods

**FIGURE 2:**
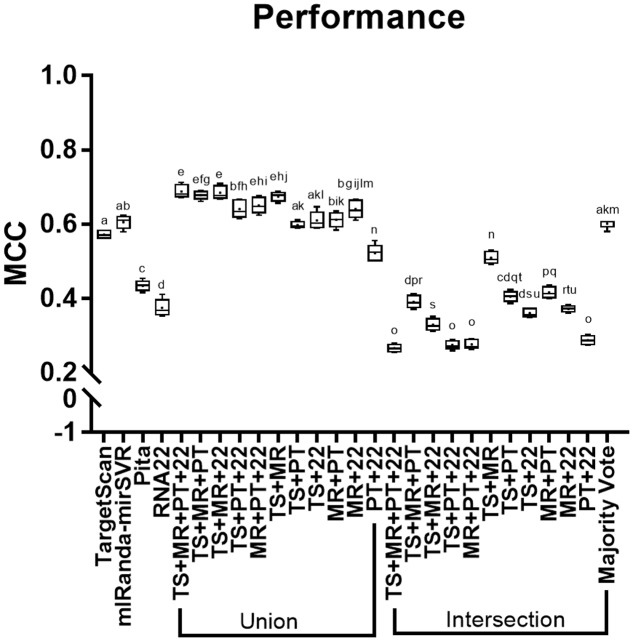
**Performance (MCC) of the methods analyzed.** TS, TargetScan; MR, miRanda-mirSVR; PT, Pita; R22, RNA22. Values with the same letter do not differ among each other.

All methods showed performance score values higher than 0, although intersections of TS + MR + PT + R22, TS + PT + R22, MR + PT + R22, and PT + R22 showed the lowest performance scores among all methods (i.e., below 0.3; **Figure 2**). Majority vote showed the best performance among all intersections, which were similar to TS and MR. MR showed slightly higher performance than TS, whereas R22 showed the lowest performance among the individual tools, followed by PT. The unions TS + MR + PT + R22, TS + MR + PT, TS + MR + R22, MR + PT + R22, and TS + MR achieved the highest performances with no statistical differences between them (see Supplementary Table S1 for a detailed data of MCC score for each individual miRNA and the combinatorial approach).

## Discussion

### Similarities and Singularities of Each Tool

The four tools predicted a considerable number of similar targets, with only a few exclusive targets. As expected, the number of correct predictions was more than those of false predictions, which indicated the elevated accuracy of the tools. However, a considerable subset of validated targets (around 18%) was not recovered in the outputs of any tool, indicating the existence of biological and/or methodological aspects that have not yet been addressed by prediction strategies and algorithms. These results also demonstrated that prediction tools have a tendency of identifying certain interactions between miRNA and target genes, but lack the ability to predict other putative interactions.

Comparison of the strategies showed that intersection of TS, MR, and PT predicted twice the number of targets than the intersection of the four tools, whereas R22 predicted the highest number of exclusive interactions. These findings are related to the similarity in features of the three seed-based and 3′UTR-specific algorithms for target prediction, especially between TS and MR than with R22. Moreover, most of the targets predicted exclusively by R22 were predicted either outside the 3′UTR or they referred to non-seed based interactions, showing that the use of approaches with distinct search strategies may provide valuable information about miRNA-target interactions.

Similarly, the low number of targets predicted by PT may be due to the low number of features used by its algorithm. Also, TS, MR, and R22 use input from the human genome version hg19 (released in 02/2009), whereas PT uses an older version (hg18, released in 03/2006). This may also explain the lower sensitivity of PT and demonstrates the importance of regularly updating the database.

Each tool has a unique set of learning attributes (see **Table 4** for more details); however, we noticed that for TS, MR, and PT (that focus on the 3′UTR) the missing features in one tool appeared after an update. For instance, the most recent update of TS (TargetScan 7.1, 2016) uses 16 features that are considered important for miRNA target recognition, which generates a score called the “Weighted Context ++ Score” (WCS). The mirSVR score (version 3.3a, 2010) is a new ranking system that scores targets predicted by miRanda using seven features to improve the MR approach. PT was last updated in 2008 (version 6) and considers only five parameters to perform the target prediction. R22 (version 2, 2015), which focuses on the entire transcript and full-length matches, uses a completely different subset of features, which may explain the differences in the targets identified by R22 and the other tools.

**Table 4:** Summary of the learning attributes of each tool.

Groups	Attributes	TargetScan	miRanda-mirSVR	Pita	RNA22
Duplex features	Seed match	X	X	X	X
	3′ contribution	X	X	X	X
	SPS	X			
	Heteroduplex free energy				X
	*p*-value				X
Local context features	SA	X	X	X	
	Flanking AU	X	X	X	
Global context features	TA	X			
	Paring position	X	X		
	3′UTR length	X	X		
	Sequence length				X
	Conservation	X	X	X	
–	Others	X			X


*SA, site accessibility; TA, transcriptome abundance.*

Individually, MR showed the best performance with the best balance between sensitivity, specificity, and precision, thus making it the optimal individual choice in most cases. However, TS and PT showed better precision with the lowest number of false positives. Thus, they could also be used if the objective is to select only few target genes for validation. In this case, TS provides a larger amount of predicted genes that can be selected for further analysis than PT. R22 showed inferior performance compared to those of other tools owing to its slightly lower specificity and precision. However, R22 is a unique tool that takes into account non-seed based matches and sites outside the 3′UTR, making it a valuable choice for searching putative non-canonical interactions. It is noteworthy that this analysis is somewhat limited by the number of miRNAs investigated and increasing the number of miRNAs might give a more comprehensive picture of miRNA-target predictions.

Throughout the analysis, all tools demonstrated both positive and negative aspects (summarized in **Table 5**). For instance, TS has a practical and user-friendly online database, containing the highest number of species that can be analyzed among all tools. However, TS assigns the same targets for miRNAs with similar seed (miRNAs of the same family), which is a drawback considering that the 3′ region of the miRNA has an important impact on target recognition (Broughton et al., 2016). Additionally, TS does not allow users to change the parameter cutoffs neither in the online data nor in the source code. MR offers the possibility of changing input parameter cutoffs in the source code, although it is not possible to do so in the pre-computed data. However, the miRanda database is less user-friendly than TS, which causes difficulty in simultaneous visualization of several targets. Moreover, the mirSVR scores are not available in the source code. PT allows the user to manipulate input parameter cutoffs in both online data and source code. This tool also enables online predictions of user 3′UTR and miRNA queries that were not pre-computed. However, PT’s online applications do not possess any interactive view of the miRNA-target pairing, relying only on the statistical numbers of the predictions. Finally, R22 has a user-friendly database that allows predictions of distinct RNA classes and database versions. Additionally, users are allowed to manipulate input parameter cutoffs in both online data and source code, although it is possible to only filter a miRNA sequence but not an mRNA target online. The disadvantage of R22 is that its source code has an increased the run time compared to those of other tools (data not shown).

**Table 5:** Positive and negative aspects of the target prediction tools analyzed.

Tool	Positive aspects	Negative aspects
TargetScan	- Friendly user database	- Predictions are the similar for all members of a miRNA family
	- Highest number of organism available	- Not possible to change parameter cutoffs
miRanda-mirSVR	- Possible to change parameter cutoffs in source code only	- Database not so friendly
		- mirSVR score not available in source code
Pita	- Possible to change parameter cutoffs	- Not shows interactive view of miRNA-target pairing
	- Enable online predictions of users miRNA and 3′UTR	
RNA22	- Friendly user database	- Source code takes too long to run
	- Allows predictions in multiple sources	
	- Possible to change parameter cutoffs	


### Intersection versus Union

There is no consensus regarding the gold standard for miRNA target prediction. The main questions are whether a tool that is superior to the existing tools exists and whether the intersection or union of two or more tools should be used to acquire more reliable results. According to Witkos et al. (2011), mixing the results from distinct tools decreases the performance of the prediction. They also indicate that the intersection of the results from two or more tools improves specificity at the cost of decreasing sensitivity, whereas the union of two or more tools increases the number of true targets as well the number of false targets detected, which decreases the specificity. Therefore, they suggest using a single target prediction tool. However, several researchers use the intersection approach (D’Aurizio et al., 2016; Wang et al., 2016) to avoid false-positive prediction regardless of the loss in sensitivity. Therefore, the use of single tool and an intersection of distinct tools are currently the most common methods of target prediction.

Our analysis revealed that the intersection strategy showed the lowest performance. All intersections showed results that were inferior to the predictions of the individual tools (**Figure 2**). The lowest performance was obtained by intersections of PT and R22. This may be due to the low sensitivity level of PT (**Figure 1A** and **Table 2**) in addition to the differences in the true targets identified by R22 and the other tools. Thus, intersections involving these tools exclusively identify few overlapping targets. Conversely, the intersections of TS + MR and majority vote, which do not depend on PT and R22, showed better performance, although they were inferior to those of TS and MR alone.

The methods with the best performance were the unions of TS + MR + PT + R22, TS + MR + PT, TS + MR + R22, MR + PT + R22, and TS + MR, with no significant difference between them (**Figure 2**). Interestingly, all validated targets predicted by PT (with the exception of two targets) were also predicted by one of the other tools (**Figure 1A**). Thus, inclusion of PT is not required for the union approach. The main difference between the unions of TS + MR + R22 and TS + MR was in the balance of sensitivity and specificity/precision. The union of TS + MR + R22 has high sensitivity (0.82) but lower specificity and precision (0.86 for both), whereas the union of TS + MR has lower sensitivity (0.71) but high specificity and precision (0.95 and 0.93, respectively). Therefore, the choice of the best approach depends on the intended use of the target prediction output.

Researchers perform target prediction analysis for two main reasons. First, to support the subsequent experimental validation of the miRNA–mRNA interaction predicted *in silico*. Second, to select the best candidates for gene ontology enrichment analysis and to identify biological processes that require the activity of these miRNAs. Both objectives demand caution during target prediction analysis. Experimental validation of miRNAs is time-consuming and costly, and therefore, selection of correct positively predicted targets is fundamental for this functional analysis. On the other hand, the quality of gene ontology enrichment analysis strongly depends on the number of inputs. The use of low number of genes as input often does not return results since the data is too scanty to obtain statistically significant values. The TS + MR union provides greater specificity and precision levels, and is recommended for the majority of analyses related to experimental validation of target sites. The TS + MR + R22 union has greater sensitivity, and is appropriate for performing subsequent functional enrichment analysis. Additionally, the TS + MR + R22 union can detect non-canonical interactions (outside 3′UTR and/or full-length match) and is also recommended for exploratory analysis or when most of the targets of the studied miRNA have been validated (although the last option has not yet been fully accomplished). The only disadvantage of using the union of two or more tools is that the scores of these tools (WCS from TS, mirSVR from MR, and minimum free energy and *p*-value for R22) are composed of different parameters and do not correlate with each other. Therefore, this approach cannot be used if the final predictions require ranking. In such cases, a single tool should be selected according to the experimental design. For most cases, MR offers the best performance.

The poor performance of the intersection approach demonstrates the importance of sensitivity in miRNA target prediction. Until recently, target prediction tools provided outputs with hundreds of false-positive targets per miRNA, which fuelled efforts for enhancing the overall quality of predictions. However, our data shows that the last available updates of the tools have high specificity and precision levels, independent of the method used to combine the data. Thus, the new challenge is to improve the sensitivity of the analysis without decreasing specificity and precision. Since all the parameters governing miRNA-target interaction are not known, the tools use severe cutoffs in the existing parameters (e.g., no mismatch in the seed region) to eliminate false positive predictions, which results in the exclusion of several correct targets. Identification of new features involving miRNA-target recognition may allow these tools to attenuate these cutoffs and increase the range of putative true targets. For example, it is well known that the 3′UTR undergoes alternative polyadenylation (aPa), resulting in transcripts with distinct 3′UTR length in different tissues (Di Giammartino et al., 2011; Yeh and Yong, 2016), which may affect miRNA recognition and regulation. Recent studies showed that conserved miRNA sites are preferentially enriched immediately after aPa sites, and thus, 3′UTR shortening is a potential escape mechanism from miRNA-mediated regulation (Hoffman et al., 2016). TS has already considered aPa sites in its last update; however, since data for the majority of species is still scarce, researchers consider the data for only few cell types and extrapolate those results to a whole organism (Agarwal et al., 2015). In addition, certain interactions are influenced by chromosomal architecture. Considering that chromosomes reside in specific locations inside the nucleus (called chromosome territories; Cremer and Cremer, 2001, 2010) that vary among cell types (Marella et al., 2009), the miRNA-mediated regulation of a gene can fluctuate depending on the proximity of these two mature molecules in the cytoplasm. Study of these and other unknown properties of cellular and genomic parameters can improve the sensitivity of target prediction tools.

## Conclusion

Current versions of the miRNA target prediction tools evaluated in this study possess high specificity and precision, generating results with negligible false positive rate. This shows that further use of the intersection strategy to obtain high quality predictions is not required. We also found that several true targets were not identified by these tools, necessitating the union of several tools for improving sensitivity. Thus, improvement of sensitivity should be the objective of the next updates.

Overall, the unions of TS + MR, as well as that of TS + MR + R22 provided better results in miRNA target prediction in terms of higher specificity and precision, whereas the latter offers remarkable sensitivity. Therefore, we recommend using these approaches prior to designing target validation experiments. However, the union approach should be avoided when ranking of the output is required. In this scenario, MR provided the best performance.

## Author Contributions

AO, LB, NL, and DP designed the experiment. AO analyzed the data. AO, LB, PN, and MH involved in data interpretation and results discussion. AO, LB, PN, and MH wrote the first draft of the manuscript. AO, NL, and DP critically reviewed and wrote the final manuscript. All authors read and approved the final manuscript.

## Conflict of Interest Statement

The authors declare that the research was conducted in the absence of any commercial or financial relationships that could be construed as a potential conflict of interest. The reviewer EL and handling Editor declared their shared affiliation, and the handling Editor states that the process nevertheless met the standards of a fair and objective review.

## Footnotes

1 http://www.targetscan.org/faqs.Release_7.html

2 https://genie.weizmann.ac.il/pubs/mir07/mir07_data.html

3 http://mirtarbase.mbc.nctu.edu.tw
