# Supplementary material for: Combining Results from Distinct MicroRNA Target Prediction Tools Enhances the Performance of Analyses
Source: Front Genet. 2017 May 16;8:59. doi: 10.3389/fgene.2017.00059 (PMC5432626; doi:10.3389/fgene.2017.00059)
Supplement: Supplementary file 3 [file Data_Sheet_1.DOCX]

Supplementary Material

**Combining results from distinct microRNA target prediction tools enhances the performance of analyses.**

**Arthur C. Oliveira, Luiz A. Bovolenta, Pedro G. Nachtigall, Marcos Edgar Hernenhoff, Ney Lemke, Danillo Pinhal***

*** Correspondence:** Danillo Pinhal: dlpinhal@gmail.com

# Supplementary Tables

**1.1 Supplementary Table 1.** Sensitivity, specificity, precision and MCC of each tool and combinatorial approach from the five replicates. TS= TargeScan, MR = miRanda-mirSVR, PT = Pita and R22 = RNA22.

**1.2 Supplementary Table 2.** Ensembl Gene ID of the targets predicted by each tool and the targets validated for each miRNA.

# Supplementary Figure


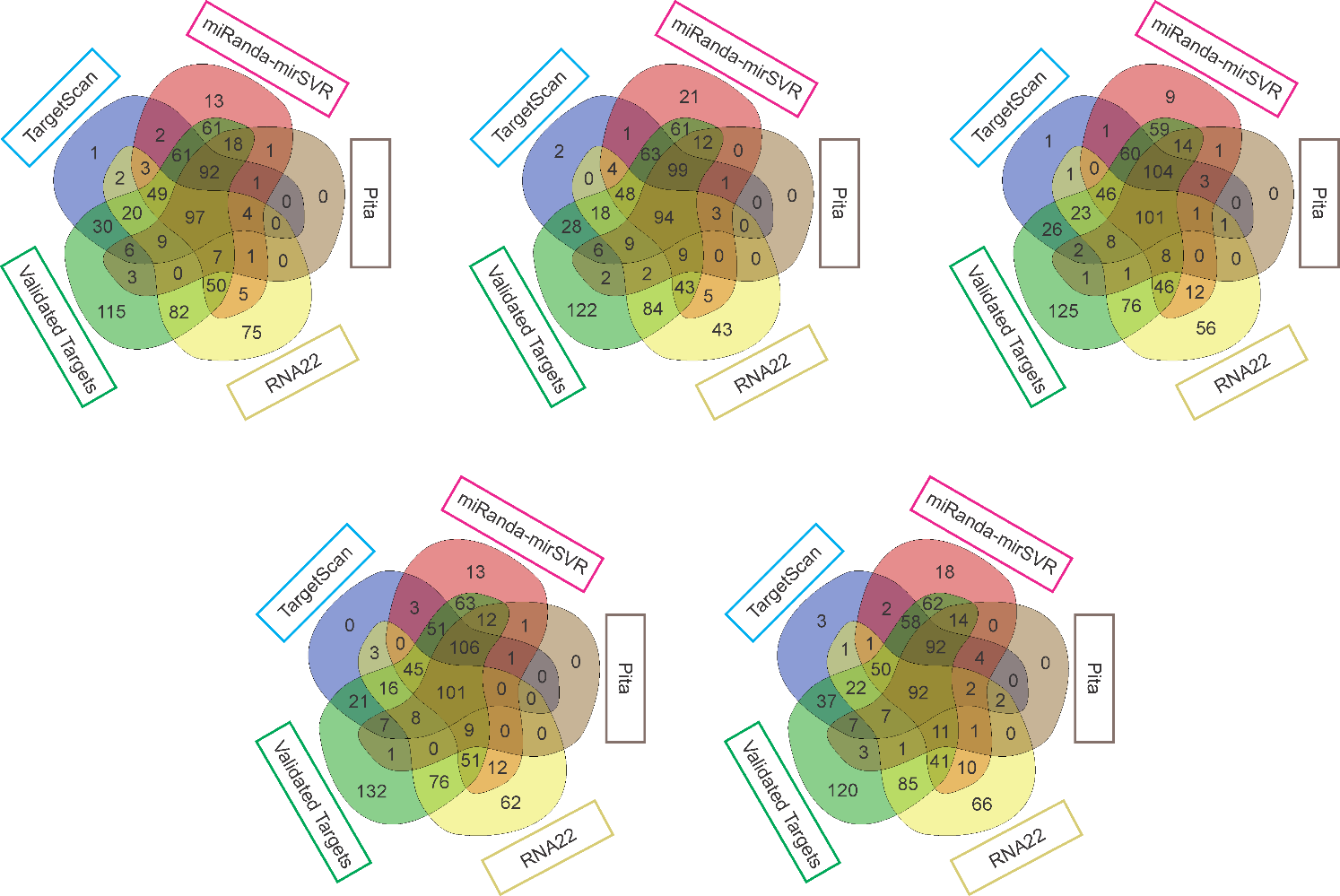


**Supplementary Figure 1.** Veen diagrams showing the number of predicted targets of each tool and combinatorial approach and the number of validated targets not predicted in each replicate.
